# Co-Treatment With Resveratrol and FGF1 Protects Against Acute Liver Toxicity After Doxorubicin Treatment *via* the AMPK/NRF2 Pathway

**DOI:** 10.3389/fphar.2022.940406

**Published:** 2022-08-30

**Authors:** Xianchou Xu, Qingbo Liu, Jiahao Li, Mengjie Xiao, Ting Gao, Xiaohui Zhang, Guangping Lu, Jie Wang, Yuanfang Guo, Peinan Wen, Junlian Gu

**Affiliations:** ^1^ Department of Breast Surgery, General Surgery, Qilu Hospital of Shandong University, Jinan, China; ^2^ Department of Gastrointestinal Surgery, Pingyang Affiliated Hospital of Wenzhou Medical University, Wenzhou, China; ^3^ School of Nursing and Rehabilitation, Cheeloo College of Medicine, Shandong University, Jinan, China

**Keywords:** doxorubicin, hepatotoxicity, AMPK, NRF2, oxidative stress

## Abstract

Doxorubicin (DOX), an anthracycline type of chemotherapy, is an effective therapy for several types of cancer, but serious side effects, such as severe hepatotoxicity, limit its use currently. Accordingly, an effective therapeutic strategy to prevent DOX-related hepatotoxicity is urgently needed. Through the inhibition of oxidative stress, fibroblast growth factor 1 (FGF1) is an effect therapy for a variety of liver diseases, but its use is limited by an increased risk of tumorigenesis due to hyperproliferation. Resveratrol (RES), a natural product, inhibits the growth of many cancer cell lines, including liver, breast, and prostate cancer cells. Therefore, this study explored whether and how RES in combination with FGF1 can alleviate DOX-induced hepatotoxicity. The results showed that RES or FGF1 alone improved DOX-induced hepatic inflammation, apoptosis and oxidative stress, and these adverse effects were further attenuated after treatment with both RES and FGF1. Mechanistically, both *in vivo* and *in vitro* results showed that RES/FGF1 reduced oxidative stress and thereby alleviated liver injury by promoting nuclear translocation of nuclear factor erythroid 2-related factor 2 (NRF2) and subsequently upregulating expression of antioxidant proteins in an adenosine monophosphate-activated protein kinase (AMPK)-dependent manner. Together, our results not only demonstrate that co-treatment with RES and FGF1 significantly inhibited DOX-induced hepatic inflammation and apoptosis, but also that co-treatment with RES and FGF1 markedly suppressed DOX-induced hepatic oxidative stress, *via* targeting the AMPK/NRF2 pathway and subsequently ameliorating hepatic dysfunction. Thus, the combination of RES and FGF1 may provide a new therapeutic strategy for limiting DOX-induced hepatotoxicity.

## Introduction

The anthracycline doxorubicin (DOX) is a widely used chemotherapeutic drug with efficacy against a wide range of cancer types (Tacar et al., 2013). However, DOX use is severely limited in both dose and duration, because it causes harmful increases in liver fibrosis, inflammation and oxidative stress (Nagai et al., 2015; Aljobaily et al., 2020). The liver is one of the organs most severely affected by DOX side effects, because, as the hub of metabolism, it receives, accumulates, and metabolizes high concentrations of DOX during treatment. Unfortunately, DOX-induced acute liver injury is a common life-threatening condition among cancer patients that can develop into acute liver failure with high mortality. Therefore, effective treatment strategies that prevent or reduce the harmful effects of DOX on the liver during chemotherapy are urgently needed.

The mechanisms of DOX-induced hepatotoxicity are complex. Recent research has identified oxidative stress as a major mechanism of DOX-induced hepatotoxicity (Song et al., 2019). Oxidative stress caused by DOX is mainly characterized by the accumulation of reactive oxygen species (ROS) and damage to the antioxidant defense system, leading to an imbalance in the production and accumulation of ROS and ultimately hepatocyte apoptosis. Accordingly, suppression of oxidative stress may be a potent strategy for attenuating DOX-induced hepatotoxicity.

Fibroblast growth factor 1 (FGF1), a well characterized mitogen, has potent anti-oxidative properties (Sun et al., 2021). Because FGF1 can promote the differentiation and maturation of liver-derived stem cells, it has been extensively studied for its therapeutic benefits in liver diseases based on the inhibition of oxidative stress (Wang et al., 2019a; Xu et al., 2020; Lin et al., 2021). Emerging evidence has revealed that the ability of FGF1 to inhibit hepatic oxidative stress is achieved *via* activation of adenosine monophosphate-activated protein kinase (AMPK), which then activates nuclear factor erythroid 2-related factor 2 (NRF2)-mediated antioxidative pathways (Lin et al., 2021). Previous research has shown that FGF1 binds to specific cell surface tyrosine kinase receptors and activates intracellular signaling, which leads to the proliferation and differentiation of multiple cell types (Delmas et al., 2016; Babina and Turner, 2017; Kostas et al., 2018). However, *in vivo* use of FGF1 is limited due to its strong mitotic activity, which leads to an increased risk of tumorigenesis with long-term use (Gasser et al., 2017; Wang et al., 2019a; Wang et al., 2021). Various natural products have been shown to inhibit tumor occurrence and metastasis without causing any toxicity or adverse side effects in many types of cancer (Zhang et al., 2018; Spradlin et al., 2019; Lin et al., 2020; Zhang et al., 2020). Thus, it is possible that the mitogenic function of FGF1 can be reduced by a natural product, combination therapy including a natural product with FGF1 might be able to facilitate the clinical application of FGF1 while minimizing the risk of cancer.

Resveratrol (RES), a natural antioxidant and free radical scavenger, is able to prevent or slow the progression of malignant tumors and is extremely effective for ameliorating oxidative stress-induced diseases (Gong et al., 2020; Izzo et al., 2021). Current preclinical evidence indicates that RES is effective at reducing alanine aminotransferase (ALT) and hepatic steatosis in patients with non-alcoholic fatty liver disease and preventing metabolic syndrome related to high-fat feeding (Faghihzadeh et al., 2015; Zhao et al., 2019a; Movahed et al., 2020). Very recently, a randomized clinical trial involving 13 patients with type 1 diabetes reported that RES supplementation exerted strong antidiabetic and antioxidant effects (Movahed et al., 2020). Previous studies have demonstrated that RES reduces the incidence of hepatoma by restoring cellular antioxidant defenses and increasing NRF2 expression in the liver (Ko et al., 2017; Izzo et al., 2021). NRF2 is one of the major regulators of the antioxidant defense system and an indirect target of RES (Xia et al., 2017). A recent study showed that RES can attenuate non-alcoholic fatty liver disease through NRF2 signaling (Hosseini et al., 2020). Additionally, RES has been shown to protect cardiomyoblasts from DOX-induced apoptosis *via* activation of AMPK (Liu et al., 2016). Based on these prospective findings, we hypothesized that RES may exert protective effects against the hepatotoxicity induced by DOX by inhibiting the proliferative activity of FGF1 and enhancing its antioxidant capacity. Therefore, the aim of the present study was to investigate whether co-treatment with RES and FGF1 could protect against DOX-induced hepatocyte injury and to determine the roles of AMPK and NRF2 in the mechanism of this potential hepato-protective effect.

## Materials and Methods

### Animals and Treatments

Eight-week-old C57BL/6J male mice were purchased from Vital River Laboratories (Beijing, China) and housed in the controlled environment at 22°C with a 12-h light/12-h dark cycle and free access to rodent chow and tap water. All mice were acclimatized for 1 week before experiment. All experimental procedures involving animals were approved by the Animal Care and Utilization Committee of Shandong University. For the animal study, C57BL/6J male mice were randomly divided into five groups (*n* = 6 per group): 1) Control group (Ctrl); 2) DOX treatment group (DOX); 3) DOX plus RES treatment group (D + R); 4) DOX plus FGF1 treatment group (D + F); and 5) DOX plus RES and FGF1 co-treatment (D + R + F). RES (10 mg/kg/day) and FGF1 (0.5 mg/kg/day) or the same volume of vehicle (0.9% saline) were intraperitoneally injected every day for 7 days. After that, mice were given a single intraperitoneal injection of DOX (20 mg/kg) or vehicle (0.9% saline). All mice in each group were euthanized at 24 h after the DOX injection. The liver was removed and weighed, and the liver index (liver weight/body weight×100%) was calculated.

### Cell Culture and Treatments

Primary mouse hepatocytes were isolated from the C57BL/6J mice by *in situ* digestion under aseptic conditions as previously described (Lin et al., 2021) and cultivated with William’s E Medium (WE) medium supplemented with 5% fetal bovine serum (Gibco, Grand Island, NY, United States), 100 U/ml penicillin and 100 μg/ml streptomycin in a humidified incubator containing 5% CO_2_ at 37°C until subsequent analysis. Primary hepatocytes were transfected with negative control-shRNA (NC-shRNA), *Ampk*-shRNA, or *Nrf2*-shRNA using a transfection reagent from Obio Technology (Shanghai, China) at 70%–90% confluency according to the manufacturer’s instructions. Primary hepatocytes were treated with RES (20 μM) and/or FGF1 (100 ng/ml) in the presence or absence of DOX (1 μM) for 24 h at 37°C. Then cells were collected to observe the level of oxidative stress after knockdown of *Ampk* or *Nrf2*.

Human hepatocellular carcinoma (HepG2) cells were cultured in high-glucose Dulbecco’s Modified Eagle’s Medium (DMEM, Macgene, Beijing, China) supplemented with 10% fetal bovine serum (Gibco), 100 U/ml penicillin and 100 μg/ml streptomycin in a 5% CO_2_ incubator at 37°C. HepG2 cells were planted at a density of 3 × 10^3^ cells/well in 96-well plates and then treated with RES (20 μM) and/or FGF1 (100 ng/ml) in the presence or absence of DOX (1 μM). After 24 h, cell proliferation was determined using a Cell Counting Kit-8 (CCK-8, Beyotime Biotechnology, Shanghai, China) according to the manufacturer’s instructions.

### Biochemical Analysis of Serum

Serum samples were acquired from mice of every group and stored at −80°C for subsequent analysis. To assess the extent of liver injury, serum ALT and aspartate aminotransferase (AST) were detected using commercial kits (Nanjing Jiancheng Biological Engineering Institution, Nanjing, China) following the manufacturer’s instructions.

### Histological and Cellular Staining

For staining analysis, mouse liver tissues were isolated, fixed in 10% formalin, and embedded in paraffin. After deparaffinization and rehydration, the paraffin sections (5 μm thick) were subjected to hematoxylin and eosin (H&E) staining, immunohistochemical (IHC) staining, or immunofluorescent (IF) staining. Sections stained with H&E (Servicebio Technology, Wuhan, China) were utilized to evaluate the hepatic histological morphology as described previously (Aljobaily et al., 2020). IHC staining with anti-tumor necrosis factor-α (TNF-α, 1:300, Abcam, Cambridge, UK), anti-3-nitrotyrosine (3-NT, 1:300, Millipore, Billerica, MA, United States), and anti-4-hydroxynonenal (4-HNE, 1:300, Abcam) as well as IF staining with anti-heme oxygenase-1 (HO-1, 1:200, Proteintech, Chicago, IL, United States) and anti-NAD(P)H quinone dehydrogenase 1 (NQO1, 1:50, Santa Cruz Biotechnology, Santa Cruz, CA, United States) were performed as described previously (Aljobaily et al., 2020).

To assess apoptosis among liver cells, terminal deoxynucleotidyl transferase dUTP nick-end labeling (TUNEL) staining was applied to tissue sections using an *In Situ* Cell Death Detection Kit from Sigma-Aldrich (St. Louis, MO, United States), and nuclei were counter-stained with DAPI (Abcam). To evaluate the ROS content in the liver tissue and primary hepatocytes, sections and cells were stained with a dihydroethidium (DHE) fluorescence kit (Beyotime Biotechnology), according to standard methods. For DHE staining and TUNEL staining, frozen liver tissues were fixed in 4% paraformaldehyde for 20 min. Stained sections or cells were observed with a light microscope (Nikon, Tokyo, Japan) or a fluorescence microscope (Nikon), and the results were quantified using Image J software (National Institutes of Health, Bethesda, MD, United States).

### Quantitative Real-Time Reverse Transcription Polymerase Chain Reaction (qRT-PCR)

Total RNA was extracted from liver tissues or primary hepatocytes with TRIzol reagent (Cwbio, Jiangsu, China). A HiFiScript cDNA Synthesis Kit (Cwbio) was used for the reverse transcription of RNA. The cycling protocol involved an initial denaturation step at 95°C, followed by 40 cycles at 60°C. The primers for mouse catalase (*Cat*), mouse superoxide dismutase (*Sod*), mouse *Ho-1*, mouse *Nqo1*, mouse interleukin-6 (*Il6*), mouse interleukin-1β (*Il1b*), mouse *Tnfa* and mouse glyceraldehyde-3-phosphate dehydrogenase (*Gapdh*) were purchased from Sangon Biotech (Shanghai, China). The expression levels of the target genes were normalized to that of *Gapdh*.

### Western Blot Analysis

Target protein expression was analyzed by western blotting. Liver tissues or primary hepatocytes were homogenized in RIPA lysis buffer (Beyotime Biotechnology) supplemented with protease and phosphatase inhibitors (Beyotime Biotechnology) on ice. Protein concentrations were determined using a BCA kit (Beyotime Biotechnology). The samples mixed with loading buffer were heated at 95°C for 10 min and then subjected to electrophoresis on a 10% sodium dodecyl sulfate (SDS)-polyacrylamide gel electrophoresis (PAGE) gel and electrotransferred to a nitrocellulose membrane (GE Healthcare Life Sciences, Chicago, IL, United States). After blocking with 5% nonfat milk for 1 h, the membranes were incubated in primary antibody solution overnight at 4°C. The second antibody was added the next day and incubated at room temperature for 1.5 h. The probed proteins were visualized using an enhanced chemiluminescence detection kit (Millipore) and analyzed using Image Quant 4.2 software (Tanon, Shanghai, China).

### Statistical Analysis

Data are presented as mean ± standard deviation (SD). The statistical significance of differences among groups was determined using one-way analysis of variance (ANOVA) or two-way ANOVA, followed by post-hoc pairwise comparisons using Tukey’s test in GraphPad Prism 8.0 as appropriate. Differences were considered significant if *p* < 0.05.

## Results

### Co-treatment With RES and FGF1 Attenuates DOX-Induced Liver Damage

**FIGURE 1:**
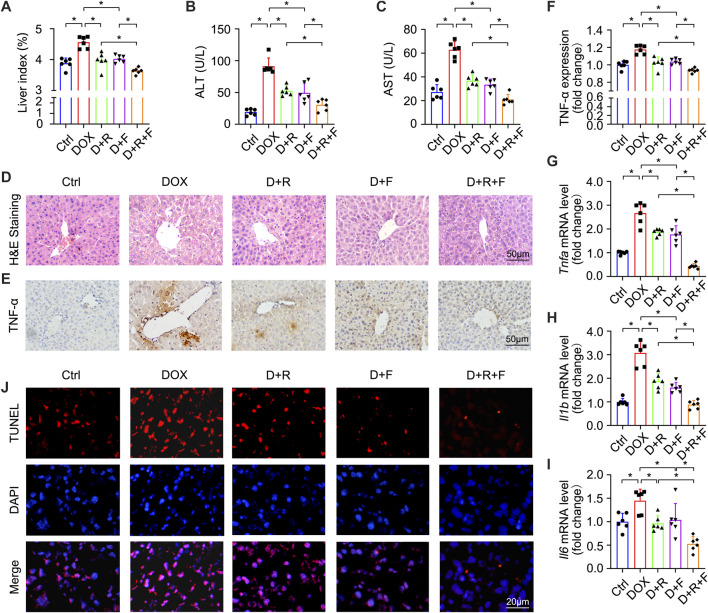
Resveratrol (RES) and fibroblast growth factor 1 (FGF1) co-treatment alleviated liver damage, inflammation, and hepatocyte apoptosis in doxorubicin (DOX)-treated mice. **(A)** Effects of RES and/or FGF1 on the liver index in mice with DOX-induced liver damage (*n* = 6). **(B,C)** Serum levels of ALT and AST after the indicated treatments (*n* = 6). **(D)** Representative light microscopy images showing histopathological changes in liver tissues stained with H&E staining. **(E,F)** Representative microscopy images showing IHC staining of TNF-α in liver tissues (*n* = 6). **(G–I)** Relative mRNA expression levels of *Tnfa*, *Il1b*, and *Il6* in hepatocytes (*n* = 6). **(J)** Representative fluorescence microscopy images of apoptotic hepatocytes labeled by TUNEL staining. Data are expressed as mean ± SD. *p < 0.05.

In order to test whether the RES and/or FGF1 treatment could affect the efficacy of DOX for inhibiting tumor cell proliferation, we utilized the CCK-8 assay to ascertain the viability of HepG2 cells. DOX potently inhibited the proliferation of HepG2 cells, and this effect was more pronounced in cells treated with the combination of RES and FGF1 (Supplementary Figure S1). To determine whether co-treatment with RES and FGF1 could alleviate DOX-induced liver damage, we first assessed overall liver function in DOX-treated mice by assessing serum AST and ALT levels as well as the liver index (Figures 1A–C). The liver index was significantly increased in the DOX group compared with the untreated Ctrl group, but treatment with RES or FGF1 alone significantly reduced the liver index compared with that in the DOX group. Notably, co-treatment with RES and FGF1 further lowered the liver index compared to either RES or FGF1 group (Figure 1A). Meanwhile, the levels of ALT and AST in mice were obviously increased after DOX treatment compared with levels in the Ctrl group, and these levels showed decreasing trends in the groups treated with RES or FGF1 only. ALT and AST levels were more obviously reduced in the group treated with both RES and FGF1 (Figures 1B,C).

H&E staining showed extensive hepatocyte necrosis and vacuoles in mouse liver slices after DOX treatment. Interestingly, co-treatment with RES and FGF1 led to reduced hepatocyte injury (Figure 1D). Given that inflammation plays a key role in DOX-induced hepatotoxicity (AlAsmari et al., 2021), we next tested indicators of hepatic inflammation. As shown in Figures 1E–I, TNF-α protein expression and mRNA expression of *Tnfa*, *Il1b,* and *Il6* were significantly increased in the DOX group compared with the Ctrl group. However, all of these changes were markedly reduced in the groups treated with either RES or FGF1 alone and even further reduced in the group treated with both RES and FGF1 (Figures 1E–I). DOX-induced hepatocyte apoptosis was assessed by TUNEL staining, and compared with the Ctrl group, an obvious increase in the number of TUNEL-positive cells was observed in the DOX group. This increase in apoptotic cells was reversed by treatment with RES or FGF1 and further attenuated by co-treatment with RES and FGF1 (Figure 1J).

### Co-treatment With RES and FGF1 Mitigates DOX-Induced Oxidative Stress in Liver Cell

**FIGURE 2:**
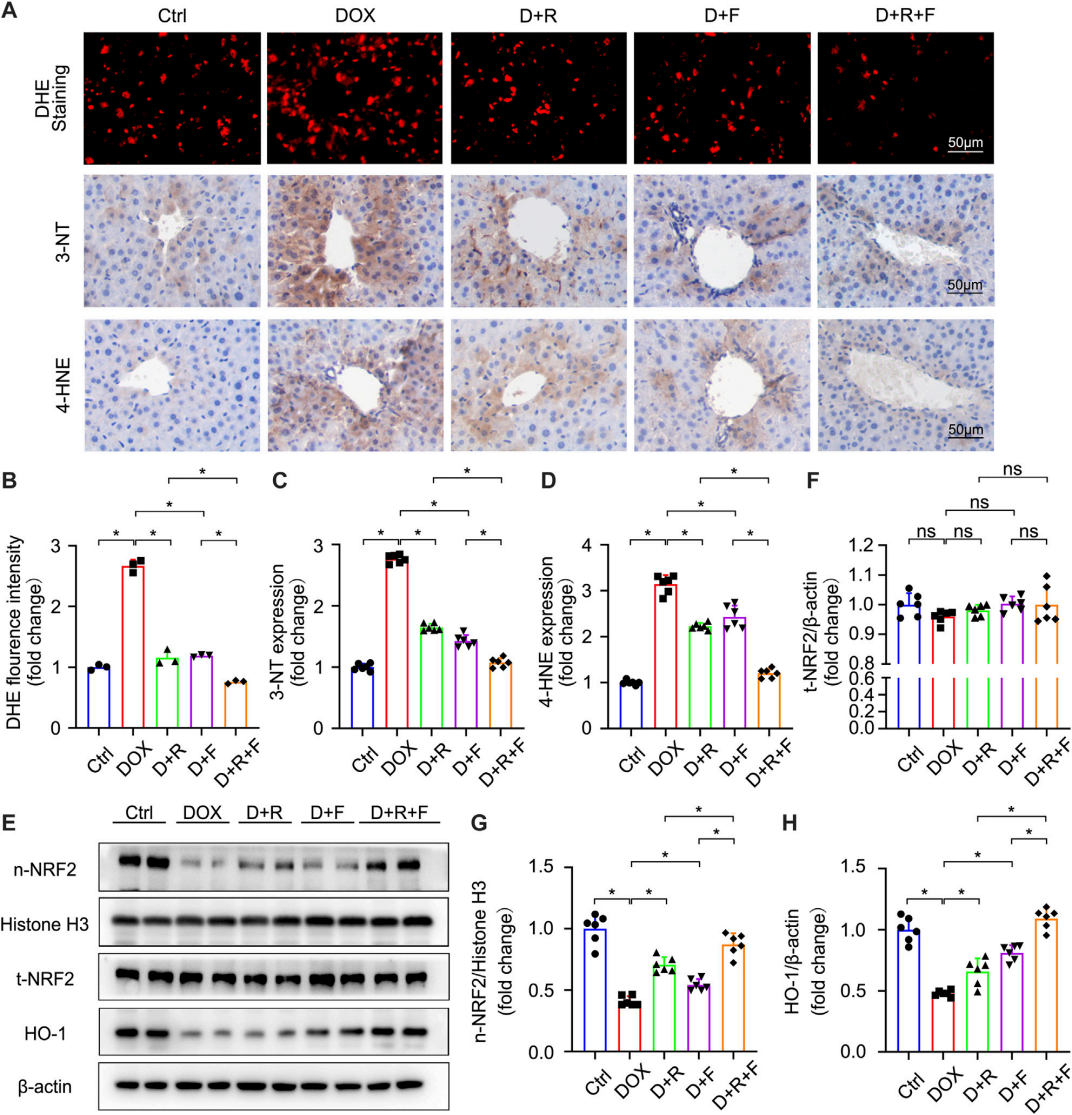
RES and FGF1 co-treatment mitigated DOX-induced oxidative stress in the mouse liver, with increases in NRF2 activity and HO-1 expression observed. **(A,B)** Representative fluorescence microscopy images of DHE-stained frozen liver tissue slices and quantitative analysis of the fluorescent intensity of DHE staining after the indicated treatments (*n* = 3). **(A,C,D)** Representative IHC staining images of **(C)** 3-NT and **(D)** 4-HNE in liver tissues, and quantification of their expression (*n* = 6). **(E–H)** t-NRF2, n-NRF2 and HO-1 protein expression. β-actin or histone H3 was used as the loading control in all western blot analysis (*n* = 6). Data are expressed as mean ± SD. *p < 0.05.

Oxidative stress results from an imbalance between the antioxidant capacity of cells and intracellular ROS levels (Farzaei et al., 2018). The liver was reported to be the most susceptible organ to DOX-induced injury and oxidative stress (AlAsmari et al., 2021). Accordingly, we next examined the ROS level in hepatocytes of the different treatment groups using the fluorescent probe DHE (Figures 2A,B). The results showed that DOX significantly increased the generation of ROS in the liver (Figures 2A,B). The levels of 3-NT and 4-HNE were also significantly increased in the DOX group based on IHC staining results (Figures 2A,C,D). These increases were obviously smaller in the RES or FGF1 only groups and further suppressed in the RES and FGF1 co-treatment group (Figures 2A–D). Taken together, these findings confirm that DOX increases oxidative stress in the liver, whereas co-treatment with RES and FGF1 can alleviate the DOX-induced increase in oxidative stress.

### NRF2 Is Required for the Protective Effect of RES/FGF1 Co-treatment Against DOX-Induced Oxidative Stress

**FIGURE 3:**
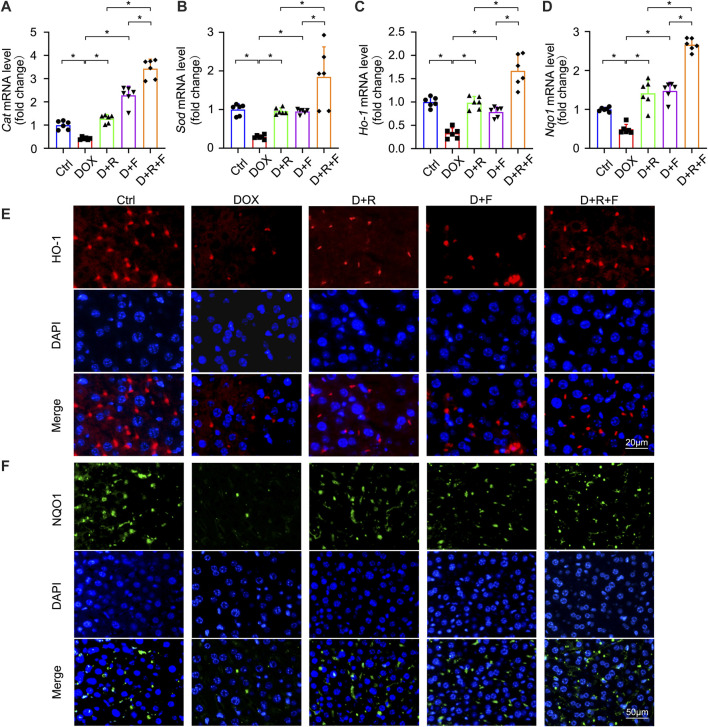
Expression of NRF2 downstream antioxidant factors CAT, SOD, HO-1, and NQO1 was elevated after RES and FGF1 co-treatment in mice. **(A-D)** Relative mRNA expression levels of *Cat*, *Sod*, *Ho-1* and *Nqo1* after the indicated treatments (*n* = 6). **(E,F)** Representative fluorescence microscopy images of IF staining for HO-1 (Red) and NQO1 (Green) in liver tissues. Data are expressed as mean ± SD. *p < 0.05.

NRF2 is the most important regulatory element in the antioxidant reaction, mediating the functions of multiple antioxidant proteins, such as HO-1, NQO1, CAT, and SOD (Ma et al., 2020). To investigate the potential involvement of NRF2 activation in the protective effect of RES and/or FGF1 against DOX-induced oxidative stress, we evaluated the effects of RES with or without FGF1 on NRF2 nuclear translocation and total NRF2 (t-NRF2) by western blot analysis (Figures 2E–G). RES previously was shown to stimulate the expression of HO-1, an important molecule in the NRF2 signaling pathway (Rao et al., 2015), and this activation is related to antioxidant effects (Kim et al., 2014). Thus, we also detected the protein expression of HO-1 (Figures 2E,H). Western blot analysis showed that nuclear translocation of NRF2 protein and HO-1 protein expression were decreased in tissues after treatment with DOX. RES or FGF1 treatment significantly ameliorated these changes, and the combination of RES and FGF1 led to further improvements compared with RES or FGF1 treatment alone (Figures 2E–H). In addition, as expected, the mRNA levels of antioxidant factors *Cat, Sod*, *Ho-1,* and *Nqo1* were decreased after DOX treatment compared with levels in the Ctrl group, and these effects were reversed by treatment with RES or FGF1 only (Figures 3A–D). More importantly, co-treatment with RES and FGF1 further reduced oxidative stress based on changes in these indicators. The IF staining of HO-1 and NQO1 further confirmed the above findings (Figures 3E,F). Taken together, these data indicate that activation of NRF2 signaling may be an important underlying mechanism for the effects of co-treatment with RES and FGF1 against hepatic oxidative stress.

**FIGURE 4:**
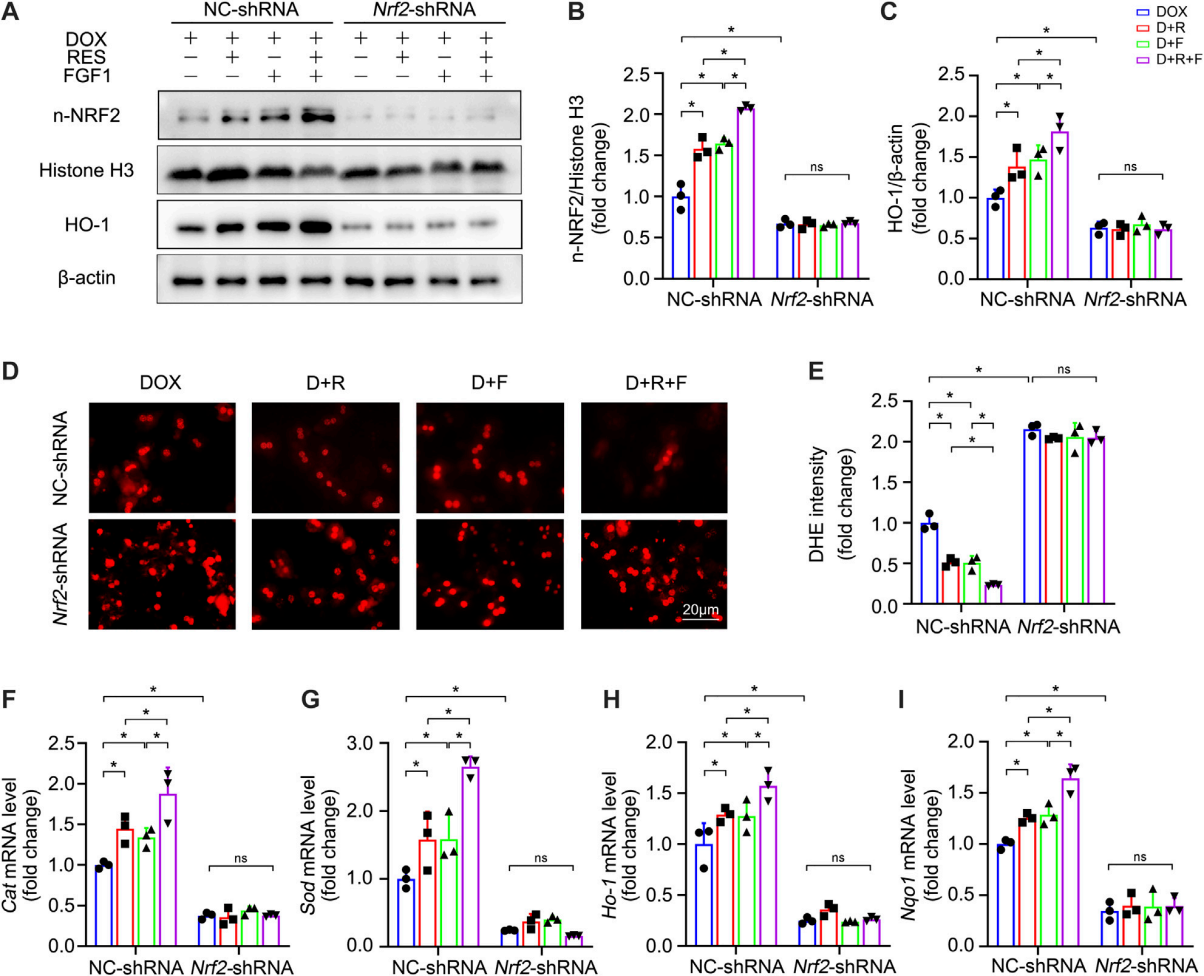
shRNA-mediated *Nrf2* knockdown diminished the protective effects of RES and FGF1 co-treatment against DOX-induced oxidative stress in primary hepatocytes. **(A-C)** Relative protein expression levels of n-NRF2 and HO-1 in primary hepatocytes after the indicated treatments. Three independent experiments were performed. β-actin or histone H3 was used as the loading control. **(D,E)** Representative fluorescence microscopy images of DHE-stained primary hepatocytes after the indicated treatments (*n* = 3). **(F–I)** Relative mRNA expression levels of oxidative stress markers *Cat*, *Sod*, *Ho-1,* and *Nqo1* in primary hepatocytes after the indicated treatments (*n* = 3). Data are expressed as mean ± SD. *p < 0.05; ns indicates not significant.

To confirm whether the antioxidant actions of NRF2 are required for the effectiveness of RES/FGF1 co-treatment in ameliorating hepatic oxidative stress, we used *Nrf2*-shRNA to knockdown *Nrf2* expression to perform loss-of-function studies in primary hepatocytes. *Nrf2* knockdown was accompanied by marked reductions in the protein levels of nuclear NRF2 (n-NRF2) and the NRF2 downstream target HO-1 (Figures 4A–C). Strikingly, knockdown of *Nrf2* completely abolished the protective effects of RES and/or FGF1 against DOX-induced oxidative stress and increased the severity of the oxidative stress response to DOX in primary hepatocytes (Figures 4D,E). Moreover, the mRNA levels of *Cat*, *Sod*, *Ho-1,* and *Nqo1* were significantly increased in primary hepatocytes after RES/FGF1 co-treatment compared with levels in the DOX group, but this effect was largely absent in cells transfected with *Nrf2*-shRNA (Figures 4F–I). These findings suggest that NRF2 plays an anti-oxidative stress role in hepatocytes by regulating the expression of antioxidant factors such as HO-1, and thus, plays a critical role in the protective effects of RES/FGF1 combination therapy in the DOX-treated liver.

### Protective Effect of RES/FGF1 Co-treatment Against DOX-Induced Oxidative Stress in Primary Hepatocytes Involves Activation of the AMPK/NRF2 Pathway

**FIGURE 5:**
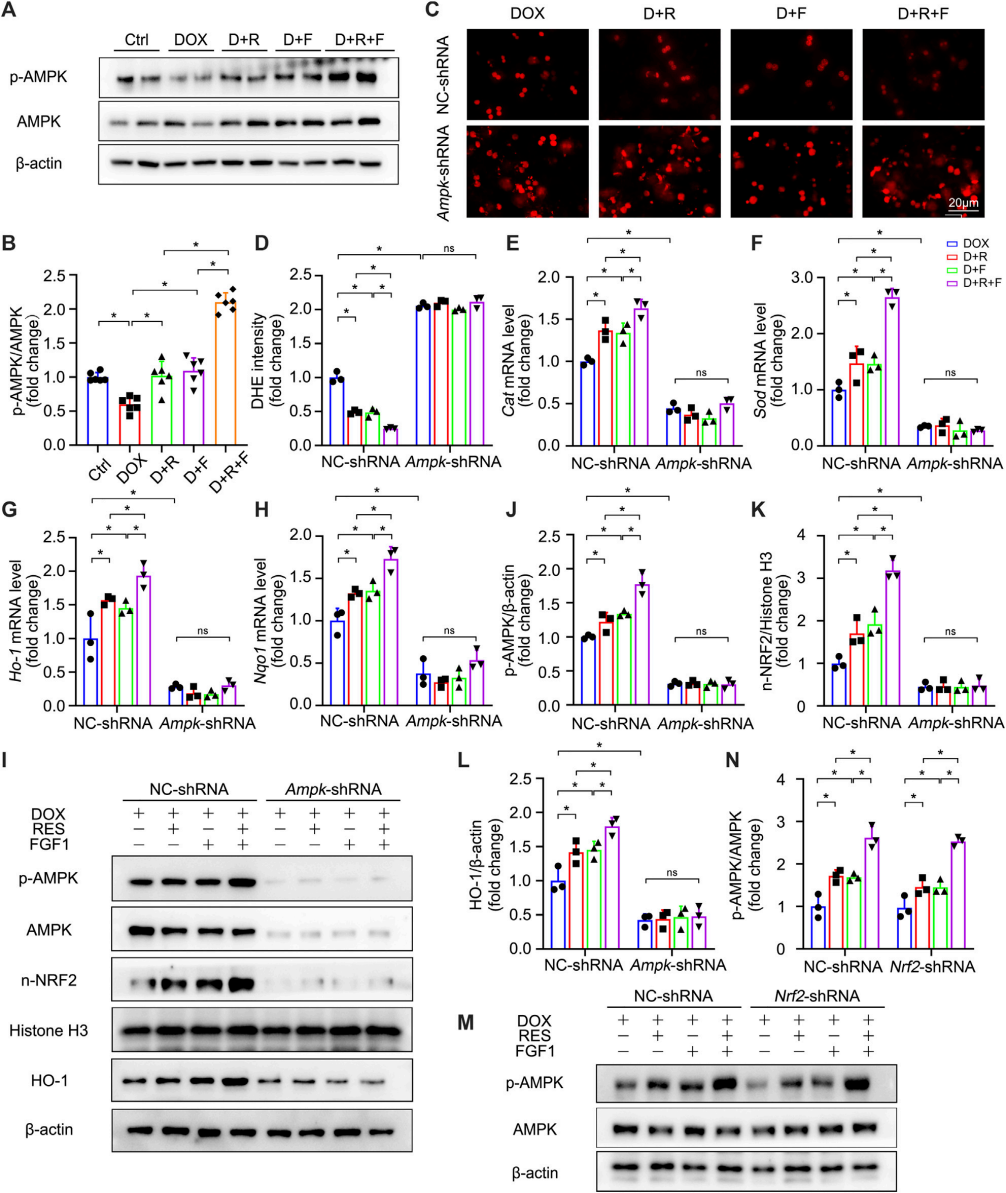
AMPK was identified as a positive upstream regulator of NRF2, which was activated upon co-treatment of primary hepatocytes with RES and FGF1. **(A,B)** Relative protein expression levels of p-AMPK and AMPK and quantification of their expression (*n* = 6). **(C,D)** Representative fluorescence microscopy images of DHE-stained primary hepatocytes of the indicated groups (*n* = 3). **(E–H)** Relative mRNA expression levels of oxidative stress markers *Cat*, *Sod*, *Ho-1,* and *Nqo1* in primary hepatocytes after the indicated treatments (*n* = 3). **(I-L)** Relative protein expression levels of proteins involved in AMPK signaling (p-AMPK/β-actin) and NRF2 signaling (n-NRF2 and HO-1) after transfection of primary hepatocytes with *Ampk*-shRNA, and quantification of their levels. **(M,N)** Relative protein expression levels of p-AMPK and AMPK in primary hepatocytes of the different groups after transfection with *Nrf2*-shRNA, and quantification of their levels. Three independent experiments were performed. β-actin or histone H3 served as the loading control for all western blot analysis. Data are expressed as mean ± SD. *p < 0.05; ns indicates not significant.

We further explored the upstream pathways leading to NRF2 activation upon RES/FGF1 co-treatment. Previous research has suggested that activation of AMPK in the liver may have important therapeutic effects in the treatment of liver diseases (Garcia et al., 2019). Activation of AMPK/NRF2 signaling molecules was also shown to protect against DOX-induced cardiomyopathy and acetaminophen-induced acute liver failure (Lv et al., 2019; Wu et al., 2020). NRF2 is controlled by AMPK, which further boosts the transcription initiation of downstream antioxidant genes of NRF2 (Qu et al., 2016; Wang et al., 2019b; Ding et al., 2020; Kong et al., 2021). Moreover, activation of the AMPKα and NRF2 pathways was shown to effectively protect the liver from cellular oxidative stress (Lee et al., 2020). Thus, we tested the role of AMPK signaling in the protection against DOX-induced hepatic oxidative stress. Western blot analysis showed that the expression of phosphorylated AMPK (p-AMPK) was decreased in DOX-treated livers, but this effect was abrogated by RES/FGF1 co-treatment (Figures 5A,B). These results suggest that the AMPK pathway is involved in protective effects of RES/FGF1 co-treatment against DOX-treated acute liver injury. Based on our results, RES/FGF1 co-treatment activated AMPK *in vivo* (Figures 5A,B), we next investigated whether AMPK plays a central role in the protective effect of RES/FGF1 co-treatment against DOX-mediated liver damage. First, we transfected primary hepatocytes with *Ampk*-shRNA, and the *Ampk* knockdown led to significantly reduced the level of p-AMPK (Figures 5I,J). *Ampk*-shRNA increased ROS production in primary hepatocytes after DOX exposure compared with the level observed in the NC-shRNA group (Figures 5C,D). qRT-PCR results revealed that the *Cat*, *Sod*, *Ho-1,* and *Nqo1* mRNA expression levels in RES and/or FGF1 groups were markedly higher than those in the DOX group; however, knockdown of *Ampk* led to lower expression levels of these indicators (Figures 5E–H). Also, n-NRF2 and HO-1 protein levels were almost completely reduced after *Ampk* knockdown (Figures 5I,K,L).

Interestingly, NRF2 activation may also influence the phosphorylation of molecules in the AMPK signaling pathway. A previous study showed that blocking the overproduction of ROS through activation of NRF2 leads to the activation of AMPK signaling, which then can alleviate excessive lipid accumulation in the liver (Chu et al., 2022). Therefore, we conducted further experiments to determine whether the protective effect of RES/FGF1 co-treatment in the liver is mediated by NRF2-induced AMPK phosphorylation. However, *Nrf2* inhibition did not change the protein expression p-AMPK (Figures 5M,N). Taken together, our results indicate that AMPK serves as a positive upstream regulator of NRF2 that is activated by co-treatment with RES and FGF1 in order to protect against DOX-induced oxidative stress.

## Discussion

The present study provides multiple lines of evidence that co-treatment with RES and FGF1 can protect against DOX-induced hepatocyte injury. Co-treatment with RES and FGF1 markedly ameliorated DOX-induced hepatic oxidative stress, and NRF2 was shown to be essential for this protective effect. Knockdown of *Ampk* expression almost completely abolished RES/FGF1-induced NRF2 activation and eliminated the anti-oxidative effects of RES/FGF1 co-treatment in primary hepatocytes. These findings reveal that RES in combination with FGF1 may offer an effective therapy for alleviating DOX-induced liver injury by reducing oxidative stress *via* the AMPK/NRF2 pathway.

As an effective antitumor drug for many cancer types, DOX has been used clinically for many years. However, its clinical utility is limited by adverse dose-dependent side effects. Studies have shown that the quinone moiety of DOX can cause severe oxidative damage to active liver substances, resulting in hepatocyte necrosis (Ma et al., 2020). The liver, as an important metabolic organ, is the most susceptible organ to DOX-induced injury and oxidative stress. Therefore, it is necessary to explore new therapeutic strategies to reduce DOX-induced hepatotoxicity. First of all, we demonstrated that treatment with the combination of RES and FGF1 enhanced the inhibitory effect of DOX on tumor cell proliferation (Supplementary Figure S1). In the present study, increases in the liver index as well as serum ALT and AST levels indicated DOX-induced liver damage. Furthermore, increased liver inflammation and hepatocyte apoptosis were observed following DOX treatment. Treatment with the combination of RES and FGF1 led to significant improvements in the above indicators (Figure 1). These findings suggest the potential effectiveness of RES/FGF1 co-treatment for protecting against DOX-induced hepatotoxicity. Although the mechanism of DOX-induced hepatotoxicity is complex, oxidative stress was proposed as one of the main mechanisms of hepatotoxicity (Vilas-Boas and Vinken, 2021). DOX causes oxidative stress in the liver and induces organ damage by inducing the production of superoxide anions and peroxynitrite radicals while decreasing the scavenging activity of O_2_^−^ (Zhao et al., 2019b). Interestingly, DOX-induced oxidative stress was significantly reduced in mice that received intraperitoneal injections with RES and/or FGF1 (Figures 2A–D). Thus, the therapeutic effect of RES/FGF1 co-treatment on DOX-induced hepatotoxicity was achieved *via* protection from oxidative stress. However, the exact molecular mechanism by which the combination of RES plus FGF1 reduces oxidative stress in the DOX-treated liver remains unknown.

NRF2 plays a critical role in resisting hepatotoxicity and is regarded as a key player in resisting oxidative stress during DOX-mediated hepatotoxicity (Barakat et al., 2018; Song et al., 2019; Ma et al., 2020). In a state of oxidative stress, ROS modifies the cysteine residues of the Kelch-like ECH-associated protein 1 protein such that it releases NRF2, which is localized in the cytoplasm. Subsequently, NRF2 enters the nucleus where it regulates the expression of downstream genes. In the present study, liver expression of NRF2 was influenced by RES/FGF1 co-treatment, which promoted NRF2 nuclear translocation; increased the expression of its downstream antioxidant factors including *Cat*, *Sod*, *Ho-1,* and *Nqo1*; and reduced ROS generation after DOX treatment (Figures 2E,G, 3). However, knockdown of *Nrf2* expression blocked the inhibitory effect of RES/FGF1 co-treatment on DOX-induced oxidative damage (Figure 4). These findings indicate that activation of NRF2 is a potent target of RES/FGF1 against DOX-induced hepatotoxicity.

NRF2 is regulated by multiple target interactions, among which AMPK plays an important role. Whether RES/FGF1 treatment influences AMPK activation and then NRF2 to counteract oxidative stress caused by DOX was further explored in this study. The level of p-AMPK was increased after RES/FGF1 co-treatment. When we blocked *Ampk* expression with *Ampk*-shRNA, not only were RES/FGF1-induced p-AMPK and n-NRF2 activities lost, but the anti-oxidative stress activities of RES/FGF1 were abolished. However, transfection of cells with *Nrf2*-shRNA did not change AMPK protein expression (Figure 5). These results verify our hypothesis that the AMPK/NRF2 signaling pathway is a critical factor in the protective effect of RES and FGF1 co-treatment against liver injury.

There are several potential limitations in our study that should be noted. First, our observations are solely based on male mouse model. Sexual dimorphism in the response to DOX treatment is due to gender differences in DOX metabolism and clearance (Grant et al., 2020; Montalvo et al., 2021). Accordingly, the pathophysiological relevance of the antioxidant and hepato-protective effects of RES/FGF1 co-treatment needs to be verified in female mice. Second, given that some studies have demonstrated how RES or FGF1 can be utilized independently to regulate AMPK, the complete signaling pathway responsible for the effects of co-treatment with RES and FGF1 requires further investigation.

**FIGURE 6:**
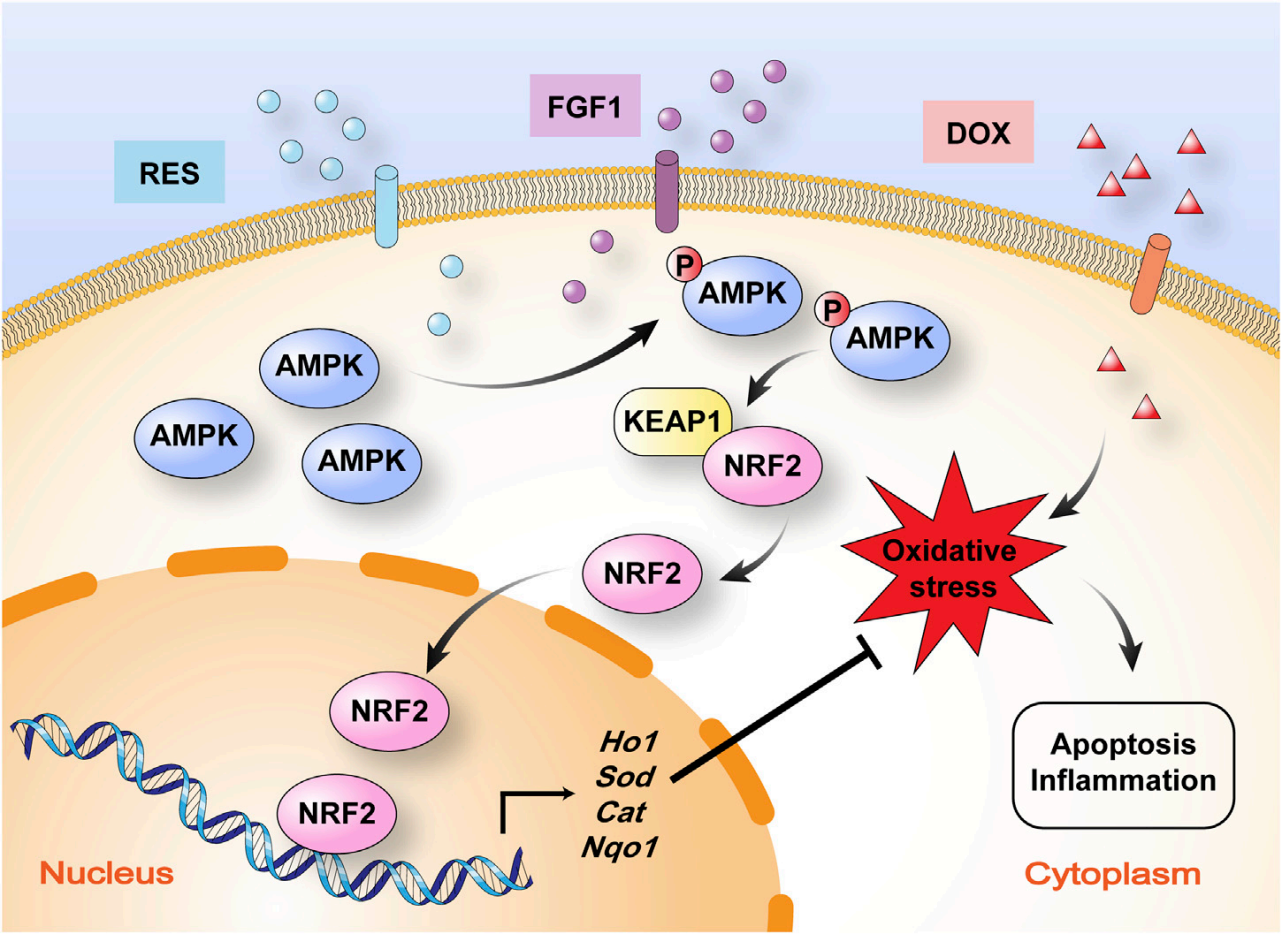
Schematic illustration of the mechanism by which RES in combination with FGF1 protects against DOX-induced hepatotoxicity. RES in combination with FGF1 increases phosphorylation of AMPK expression and nuclear translocation of NRF2 and promotes HO-1 gene expression, which alleviates DOX-induced liver oxidative stress. KEAP1, Kelch-like ECH-associated protein 1.

In summary, this study provides novel insight into a potential treatment to limit DOX-induced hepatotoxicity. We demonstrated here for the first time that RES in combination with FGF1 can alleviate DOX-induced liver injury by relieving oxidative stress *via* targeting the AMPK/NRF2 pathway, as illustrated in Figure 6. Accordingly, RES/FGF1 co-treatment could be developed as a new therapeutic strategy for improving outcomes in cancer patients receiving DOX.

## Data Availability

The original contributions presented in the study are included in the article/Supplementary Materials, further inquiries can be directed to the corresponding authors.
