# Supplementary material for: Co-Treatment With Resveratrol and FGF1 Protects Against Acute Liver Toxicity After Doxorubicin Treatment via the AMPK/NRF2 Pathway
Source: Front Pharmacol. 2022 Aug 30;13:940406. doi: 10.3389/fphar.2022.940406 (PMC9468578; doi:10.3389/fphar.2022.940406)
Supplement: Supplementary file 1 [file DataSheet1.docx]

**Supplementary materials**


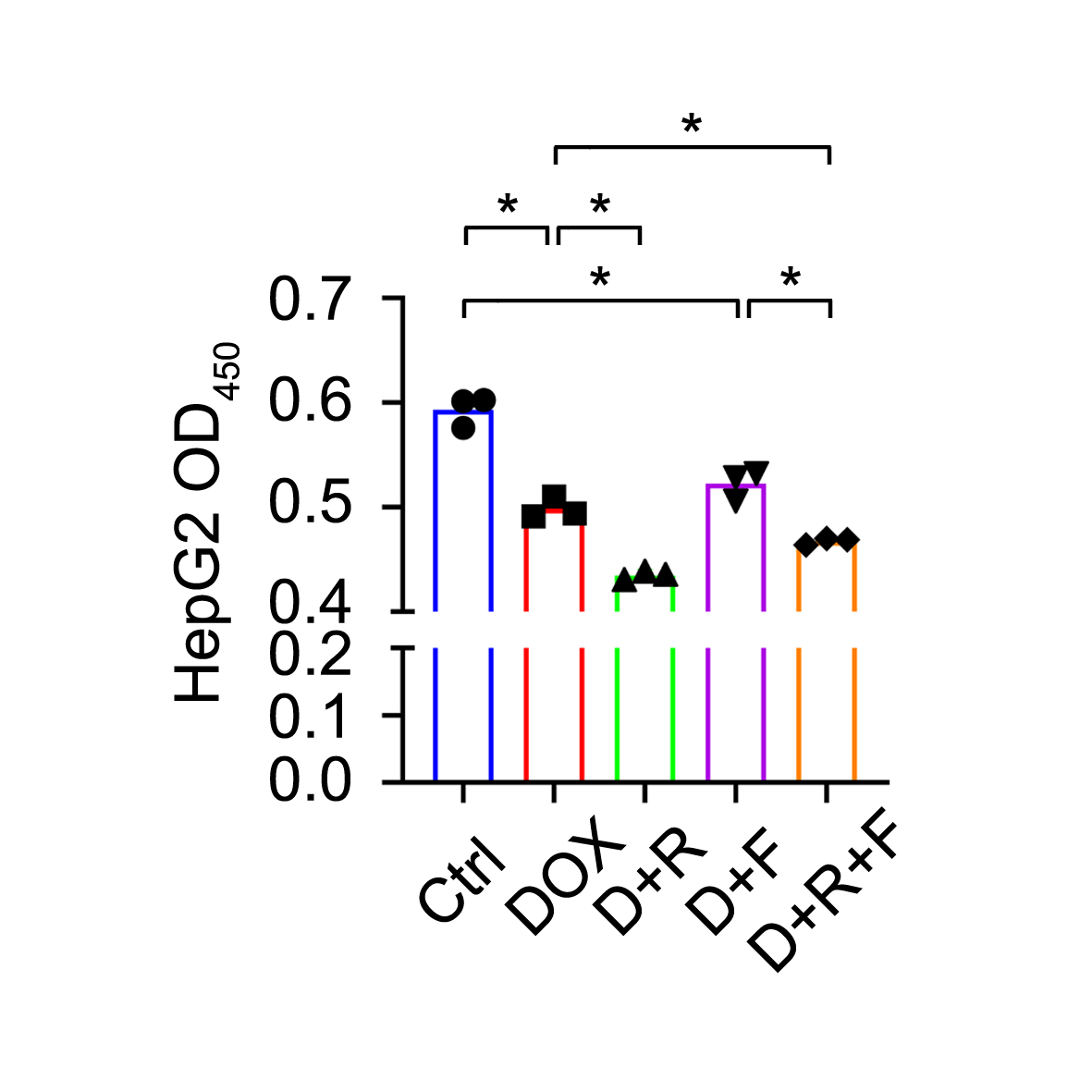


**Figure S1:** **The combination of RES and FGF1 can help enhance the effectiveness of the DOX therapy.** The cell viability was measured in HepG2 cells by CCK-8 assay (n=3). Data are expressed as mean ± SD. **P*<0.05.

| Name | Manufacturers | Production Place |
| --- | --- | --- |
| Transfection reagent | Obio Technology | Shanghai, China |
| Dulbecco’s Modified Eagle’s Medium | Macgene | Beijing, China |
| Fetal bovine serum | Gibco | Grand Island, NY, United States |
| Cell Counting Kit-8 | Beyotime Biotechnology | Shanghai, China |
| Alanine aminotransferase Assay Kit | Nanjing Jiancheng Biological Engineering Institution | Nanjing, China |
| [Aspartate aminotransferase Assay Kit](http://www.njjcbio.com/products.asp?id=779) | Nanjing Jiancheng Biological Engineering Institution | Nanjing, China |
| Hematoxylin and eosin Staining Kit | Servicebio Technology | Wuhan, China |
| Anti-tumor necrosis factor-α | Abcam | Cambridge, UK |
| Anti-3-nitrotyrosine | Millipore | Billerica, MA, United States |
| Anti-4-hydroxynonenal | Abcam | Cambridge, UK |
| Anti-heme oxygenase-1 | Proteintech | Chicago, IL, United States |
| Anti-NAD(P)H quinone dehydrogenase 1 | Santa Cruz Biotechnology | Santa Cruz, CA, United States |
| *In Situ* Cell Death Detection Kit | Sigma-Aldrich | St. Louis, MO, United States |
| DAPI | Abcam | Cambridge, UK |
| Dihydroethidium fluorescence Kit | Beyotime Biotechnology | Shanghai, China |
| RIPA lysis buffer | Beyotime Biotechnology | Shanghai, China |
| TRIzol reagent | Cwbio | Jiangsu, China |
| HiFiScript cDNA Synthesis Kit | Cwbio | Jiangsu, China |
| BCA Kit | Beyotime Biotechnology | Shanghai, China |
| Enhanced chemiluminescence detection Kit | Millipore | Billerica, MA, United States |
